# Impact of PEG Content on Doxorubicin Release from PLGA-co-PEG Nanoparticles

**DOI:** 10.3390/ma17143544

**Published:** 2024-07-17

**Authors:** Maria Margarida Cardoso, Inês N. Peça, Ana Bicho

**Affiliations:** 1 LAQV-REQUIMTE, Departamento de Química, Nova School of Science and Technology (NOVA FCT), Universidade NOVA de Lisboa, Quinta da Torre, 2829-516 Caparica, Portugal; nobre.iines@gmail.com; 2 Instituto Gulbenkian de Ciência, Rua da Quinta Grande 6, 2780-156 Oeiras, Portugal; asantos@igc.gulbenkian.pt

**Keywords:** drug-controlled release, PLGA, PLGA-PEG copolymers, doxorubicin, nanoparticles, Baker–Lonsdale model

## Abstract

Nanoparticles (NPs) have become attractive vehicles for drug delivery in cancer therapy due to their ability to accumulate in tumours and mitigate side effects. This study focuses on the production of doxorubicin (DOX)-loaded NPs comprising Poly (lactic-co-glycolic acid)-Polyethylene glycol with varying PEG proportions and the examination of their impact on drug release kinetics. DOX-loaded NPs, composed of PLGA-co-PEG with PEG contents of 0%, 5%, 10%, and 15%, were synthesized by the solvent evaporation technique, exhibited spherical morphology, and had sizes ranging from 420 nm to 690 nm. In vitro drug release studies revealed biphasic profiles, with higher PEG contents leading to faster and more extensive drug release. The Baker–Lonsdale model demonstrated the best fit to the drug release data, indicating that the release process is diffusion-controlled. The diffusion coefficients for DOX determined ranged from 6.3 × 10^−18^ to 7.55 × 10^−17^ cm^2^s^−1^ and exhibited an upward trend with increasing PEG content in the polymer. In vitro cytotoxicity tests with CHO cells showed that unloaded NPs are non-toxic, while DOX-loaded PLGA-PEG 15% NPs induced a greater decrease in cellular viability compared to their PLGA counterparts. A mathematical relationship between the diffusion coefficient and PEG percentage was derived, providing a practical tool for optimizing DOX release profiles.

## 1. Introduction

Current therapy methods for tumour treatment typically involve surgery, often followed by chemotherapy and radiation. While these treatments are effective for many forms of cancer, chemotherapy often entails significant toxicity and adverse side effects. Research into the rationale behind the delivery and targeting of therapeutic and diagnostic agents is at the forefront of projects in nanomedicine [1,2]. One of the main goals is to develop a safe and effective drug carrier for systemic application.

Nanoparticles (NPs) have demonstrated enormous potential as carriers for controlled drug delivery in cancer therapy due to their ability to accumulate in tumours post-administration and be internalized, thereby enhancing therapeutic benefits while minimizing side effects [3]. Their biodistribution is largely determined by their physical and biochemical properties, including particle size and shape, surface charge, and chemical composition [4]. Controlling the sustained release and the delivery site of a drug is crucial, and NPs offer an excellent platform for this purpose, as their size and surface properties can be readily modified to alter their behaviour towards achieving a desired goal. The drug release mechanism from a specific polymer largely depends on the physicochemical properties of the drug, its loading, and the polymer properties [5]. The degradation of particles and the release of drug profiles can be modulated by selecting appropriate matrix materials and drug concentration (which is generally high).

Various polymers, both synthetic and natural, have been utilized in formulating biodegradable NPs. Poly (D,L-lactide-co-glycolide) (PLGA), a synthetic polymer approved by the United States Food and Drug Administration (FDA), has been widely employed owing to its excellent biocompatibility, biodegradability, and mechanical strength [6]. Incorporating a hydrophilic segment such as PEG alters the physico-chemical properties of hydrophobic and biodegradable PLGA, particularly regarding its hydrophilicity and swelling, thus enhancing water uptake, which facilitates the polymer’s hydrolytic cleavage and drug release [7]. Moreover, biodistribution studies have demonstrated that NPs have an extended half-life and the capability to control the delivery of the loaded drugs [8]. Several studies on NP production using PLGA-co-PEG polymers, predominantly triblock copolymers, have been conducted [7,8,9,10,11,12]. The effect of PEG content in PEG-PLGA polymers on drug encapsulation and release has been investigated with microparticles [13,14,15,16,17], although not with nanoparticles. Since particle size has been shown to have a major influence on drug release kinetics [18,19], it is our aim to produce drug-loaded nanoparticles composed of PLGA-co-PEG copolymers with varying PEG proportions, model the drug release profile, and investigate the influence of PEG content on release the profile and mechanism. The model drug selected for this study was doxorubicin (DOX), one of the most powerful and extensively used anti-cancer agents. It functions by inhibiting nucleic acid synthesis within cancer cells, but it is connected to many adverse side effects, including cardiotoxicity and myelosuppression, contributing to its narrow therapeutic index [20]. PLGA-co-PEG NPs were analysed in relation to their shape, size, zeta potential, and crystallinity. Mechanistic and semi-empiric models were applied to describe the in vitro release data and evaluate the effect of PEG content in the polymer on drug release and mechanism. In vitro tests using cultures of mammalian cells were conducted to assess NP cytotoxicity and therapeutic potential.

## 2. Materials and Methods

### 2.1. Materials

The PLGA (50:50) (Mw 40,000–75,000), the Poly(vinyl alcohol) (PVA) (Mw 30,000–70,000) used as the emulsifier, and the doxorubicin hydrochloride were purchased from Sigma-Aldrich (St. Louis, MI, USA). The Resomer PEG type RGP (with a hydroxyl end group), d5055 (diblock, 5% PEG, Mw~600,000), d50105 (diblock, 10% PEG, Mw~700,000), and d50155 (diblock, 15% PEG, Mw~550,000) were obtained from Boehring Ingelheim (Ingelheim, Germany). The dichloromethane (DCM) with a purity of 99.9%, acetone with a purity of 95%, and phosphate buffered saline (PBS) were purchased from Fluka. The culture media, foetal bovine trypsin, antibiotics, and fungi-zone were supplied by Gibco (Billings, MT, USA), and the Live/Dead viability kit and Toto-3 were purchased from Invitrogen (Waltham, MA, USA).

### 2.2. Preparation of PLGA and PLGA-PEG Nanoparticles

The PLGA NPs or PLGA-co-PEG NPs were prepared using an oil-in-water (O/W) emulsion–evaporation method [21]. Briefly, a polymer solution containing 50 mg of polymer (PLGA or PLGA-co-PEG containing PEG contents of 5%, 10%, and 15%) in 500 µL of acetone and 750 µL of DCM was poured into 10 mL of an aqueous solution of PVA (3% *w*/*v*) that acts as a stabilizer. The mixture was emulsified by sonication for 20 s at 200 W (Model UP100H, MS2, Hielscher, Teltow, Germany). The obtained emulsion was added to 50 mL of an aqueous solution of PVA (0.25% *w*/*v*) and stirred magnetically for 24 h at room temperature to fully evaporate the DCM and harden the particles. The suspension was centrifuged at 11,000 rpm (Sartorius, Sigma 4K15, Gottingen, Germany) and the collected NPs were freeze-dried (Telstar Cryodos, Barcelona, Spain) after being washed 3 times with deionized water.

DOX-loaded NPs were prepared using the same procedure but with the addition of DOX (10 mg) to the polymeric organic solution.

### 2.3. Characterization of NPs

#### 2.3.1. Surface Morphology

The surface morphology and size of the NPs were characterized by scanning electron microscopy (SEM). The freeze-dried NPs were analysed using a scanning electron microscope (Zeiss model DSM 962, Oberkochen, Germany) after being coated with gold in an argon atmosphere. An accelerated voltage of 20 kV was used.

#### 2.3.2. Particles Size and Zeta Potential Analysis

NP size and zeta potential were measured by a Laser Light Scatter (Zetasizer Nano ZS, Malvern). The freeze-dried NPs were dispersed in an aqueous solution of PVA (0.50% *w*/*v*), sonicated, and washed 3 times with deionised water prior to the measurements (3 per sample).

#### 2.3.3. Thermal Analysis by Differential Scanning Calorimetry (DSC)

Thermal analysis of the nanoparticles (NPs) was conducted using differential scanning calorimetry (DSC) with a Setaram DSC131 (Newark, CA, USA) calorimeter provided with a thermal data analysis tool and connected to a cooling system. In total, 10 mg of NPs was sealed in aluminium pans and subjected to a heating cycle from 20 °C to 80 °C, then cooled to 25 °C, and subsequently reheated to 250 °C at a rate of 10 °C · min^−1^ under a nitrogen atmosphere.

#### 2.3.4. Drug Loading and Encapsulation Efficiency

Reverse-phase high-performance liquid chromatography (HPLC) (Hitachi-Merck, Tokyo, Japan) was used to quantify the amount of DOX in the particles using a Merck RP-18 (Merck, Darmstadt, Germany) column with an eluent composed of a mixture of acetonitrile: acetate buffer (pH 4) in a 50:50 (*v*/*v*) ratio as eluent at a flow rate of 0.8 mL min^−1^. In total, 5 mg of particles were dissolved in DCM (5 mL) and placed in a desiccator until the solvent completely evaporated, after which 2 LL of eluent were added. A UV detector (Merck Hitachi, Japan) set to a wavelength of 234 nm was utilized. The drug loading capacity was calculated as the ratio between the amount of DOX entrapped in the nanoparticles and the total amount of NPs. The encapsulation efficiency was calculated by the ratio between the drug amount in the NP and the initial weight of the drug used in NP preparation.

### 2.4. In Vitro DOX Release Studies

Centrifuge tubes containing 10 mg of DOX-loaded NPs dispersed in 7 m of PBS (pH 7.2), to assure sink conditions, were placed in a shaker set to 120 rpm at 37 °C. At set time points, the tubes were centrifuged for 16 min at 10,000 rpm and the amount of DOX in the supernatants was analysed by HPLC, as described in Section 2.3.4 (the drug loading section). The tubes were placed back in the incubator after the addition of 7 mL of fresh PBS to resuspend the precipitated NPs.

Calculation methods: Non-linear regression calculations were performed to estimate drug release parameters using the Scientist™ software (version 2) package from Micromath^®^. The least-squares and simplex algorithms were employed.

### 2.5. In Vitro Cell Tests

#### 2.5.1. Cell Cultures

The effects of nanoparticles (NPs) and DOX-loaded NPs on cell viability were evaluated in CHO cells (Hamster Chinese Ovary, ECACC No. 85050302) using a procedure previously reported [22]. Briefly, the cells were grown in Dulbecco’s modified Eagle medium (Gibco, Waltham, MA, USA) with 4.5 g/L of glucose and GlutaMAX™, 10% foetal bovine serum, penicillin (50 I.U/mL), and streptomycin (50 U.G/mL). Sub-culture was performed by trypsinization when cellular growth reached a 70% confluence. The cultures were grown on plates and incubated in a CO_2_ incubator (5% CO_2_; 37 °C) kept at 37 °C.

#### 2.5.2. Cell Viability Assays

Cell viability was assessed by cell counting and by flow cytometry as described by Peça et al. [23]. The experiments were performed using empty and DOX-containing PLGA NPs and PLGA-PEG 15% NPs. To prevent contaminations, NPs were resuspended in sterile PBS (1 mg/mL) which contain penicillin, streptomycin, and fungizone (25 µg/mL) and kept at 4 °C for up to two weeks.

- Absolute Number of Cells

CHO cells were seeded in Dulbecco’s modified Eagle medium and incubated in a CO_2_ incubator (5% CO_2_; 37 °C) for 24 h and kept at 37 °C. Following incubation, the unloaded NP or DOX-NP suspension was added. An assay with no NPs added was performed as a control. After 48 h, cells were collected, resuspended in 0.5 mL of medium, and counted using an automated cell counter (Scepter™ 2.0, Merck Millipore, Germany) with a 40 μm sensor. Scepter™ (version 2) software was used to analyse the results.

- Flow Cytometry

CHO cells were seeded in Dulbecco´s modified Eagle medium and incubated in a CO_2_ incubator (5% CO_2_; 37 °C) for 24 h kept at 37 °C. After incubation, a suspension containing NPs (1 mg NP/mL) or DOX-loaded NPs was added. An assay with no NPs added was performed as a control. After 48 h, the cells were collected and resuspended in 1 mL of medium. Unstained and single-colour controls for calcein (that identifies live cells) and Toto-3 (that identifies dead cells), as well as a sample stained with both calcein and Toto-3, were analysed for each sample. Calcein (0.2 µM) was incubated for 30 min and Toto-3 (750 µM) for 10 min, both at room temperature. A CyAn ADP (Beckman Coulter, Fort Collins, CO, USA) was used to carry out flow cytometry analysis. Lasers with wavelengths of 488 nm were applied to excite the calcein and those of 642 nm were applied to Toto-3. Calcein fluorescence was detected with a 530/30 nm bandpass filter, and Toto-3 fluorescence was detected with a 665/20 nm bandpass filter.

## 3. Results and Discussion

### 3.1. Nanoparticle Characterization

**Figure 1:**
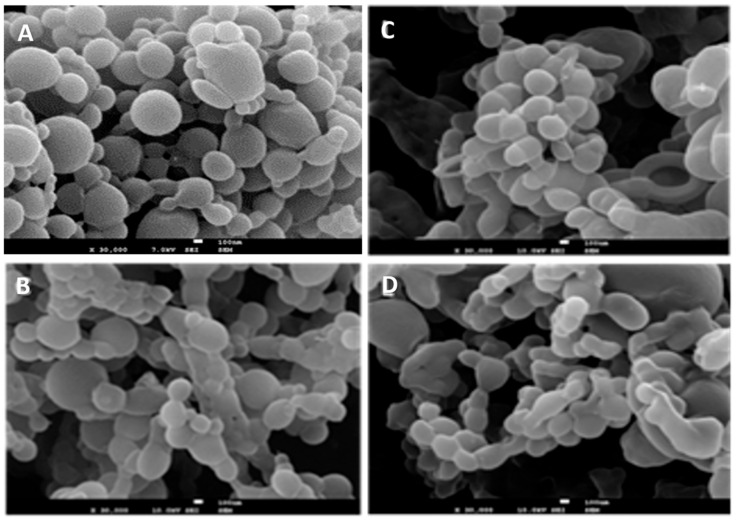
SEM images of NPs made of: (**A**) PLGA, (**B**) PLGA-PEG 5%, (**C**) PLGA-PEG 10%, (**D**) PLGA-PEG 15%. The scale bars in figures represent 100 nm.

Nanoparticles encapsulating DOX were prepared using PLGA along with three distinct PLGA-PEG copolymes: PLGA-PEG diblock with 5% PEG (PLGA-PEG 5%), PLGA-PEG diblock with 10% PEG (PLGA-PEG 10%), and PLGA-PEG diblock with 15% PEG (PLGA-PEG 15%) using an emulsion solvent diffusion method. The surface morphology of the obtained particles was characterized by scanning electron microscopy and representative images are depicted in Figure 1.

**Figure 2:**
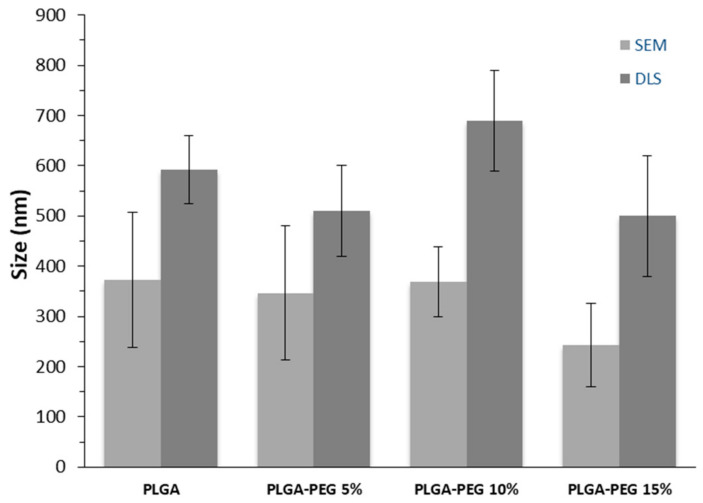
Sizes of DOX-loaded NPs of PLGA, PLGA-PEG 5%, PLGA-PEG 10%, and PLGA-PEG 15% (n = 3) determined by DLS and by SEM.

The micrographs revealed that all NPs generally exhibit a spherical shape with a smooth surface, showing no discernible differences among the various polymers. The mean particle diameters of the produced NPs obtained from dynamic light scattering (DLS) and from SEM picture measurements are depicted in Figure 2.

The sizes obtained by DLS range between 524 and 660 nm for PLGA NPs, 420 and 600 nm for PLGA-PEG 5% NPs, 590 and 790 nm for PLGA-PEG 10% NPs, and 430 and 550 nm for PLGA-PEG 15% NPs, much higher than those obtained by SEM which may indicate the presence of particle aggregates. The zeta potentials measured by DLS at a neutral pH, the drug loading, and the glass transition temperature of the NPs produced are presented in Table 1. The zeta potentials exhibit negative values, with PLGA-co-PEG NP presenting lower negative values than PLGA, which can be attributed to the replacement of the carboxylic acid groups of PLGA by PEG chains. This effect was also obtained by Khna et al. [12]. These values indicate colloidal stability for all NPs. Regarding the drug loading of NPs, defined as the percentage of DOX mass into NPs relative to the total NPs mass, similar values were obtained across all polymers, ranging from 2.6 ± 0.6 to 2.9 ± 0.6 mg of DOX/100 mg of NP. The encapsulation efficiency obtained shows low and similar values for all NPs. The DSC thermograms reveal that, like PLGA, the PLGA-co-PEG copolymers are amorphous as no melting point was observed. Amorphous polymers generally exhibit more uniform and reproducible degradation and release kinetics [24], therefore offering advantages as drug delivery carriers.

**Figure 3:**
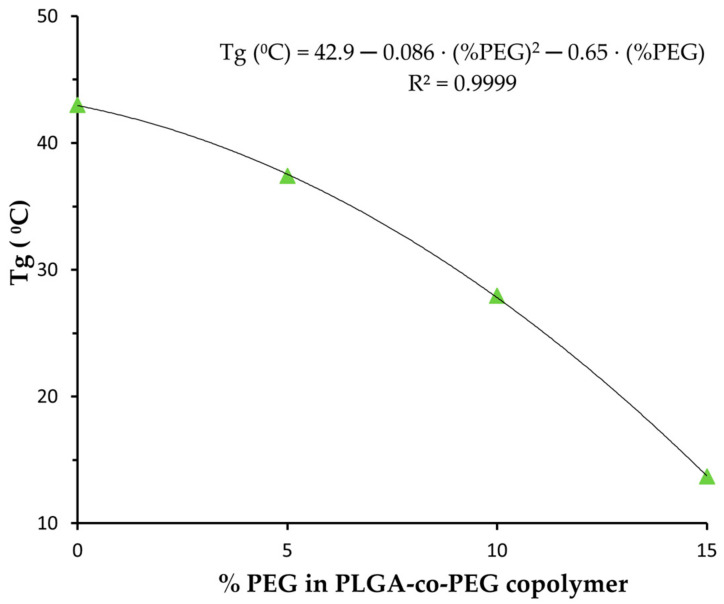
Glass transition temperature as a function of PEG content in PLGA-co-PEG NPs. The line indicates the trend equation.

Regarding the glass transition temperature, there is a great dependence on PEG content within the polymer. An increase in PEG content leads to a significant decrease in polymer Tg, indicating enhanced flexibility in polymer chains, as expected. This relationship is clearly depicted in Figure 3, where Tg is plotted as a function of PEG content in the polymers. A polynomial relation with an R^2^ of 0.9999 was obtained.

### 3.2. In Vitro Controlled Drug Release

**Figure 4:**
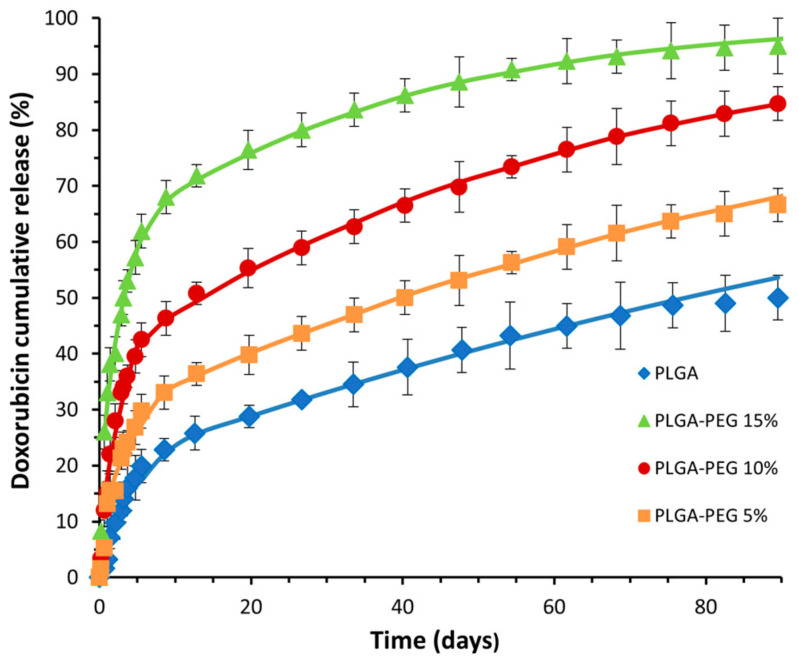
Cumulative release profiles of DOX from PLGA NP and PLGA-co-PEG NP containing 5%, 10%, and 15% of PEG, represented as the percentage of DOX released over time. The solid lines represent the fitting obtained by the Baker–Lonsdale model (Equations (1) and (2)).

To investigate the impact of PEG content in the polymer on the drug release profile, in vitro drug release experiments were conducted using NPs composed of PLGA and PLGA-PEG copolymers. The cumulative amount of drug released over time was measured, and the results for PLGA, PLGA-PEG 5%, PLGA-PEG 10%, and PLGA-PEG 15% NPs are depicted in Figure 4. The cumulative in vitro release of DOX reveals biphasic release profiles for all NPs in accordance with those obtained by other authors for PLGA-co-PEG NPs [25,26].

These profiles are characterized by an initial rapid release phase attributed to the release of DOX adhered to the surface and localized near the surface of the NPs, resulting in a short diffusion path. After day 6, release rates gradually decreased. In Figure 4, it can be observed that doxorubicin may be released faster from PLGA-containing PEG NPs compared to PLGA NPs. The extent of drug release was highly dependent on the PEG composition, with complete release occurring earlier with a higher PEG content in the polymers. The amount of doxorubicin released from PLGA NPs after 2, 12, and 60 days was approximately 10%, 25%, and 45%, respectively. In contrast, the amount of DOX released from PLGA-co-PEG NPs was approximately 16%, 36%, and 59% for PLGA-PEG; 5%; 28%, 50%, and 76% for PLGA-PEG 10%; and 40%, 71%, and 92% for PLGA-PEG 15%.

- Model Fitting

The in vitro drug release data obtained were fitted to mechanistic and semi-empirical models and the superior fitness was assessed by comparing the adjusted R^2^ square (R^2^adj) and the model selection criterion (MSC) values. 

The release profile was best explained by the Baker–Lonsdale model (fitting curves presented in Figure 4), as this mathematical modelling demonstrated the highest R^2^adj and MSC values (0.9995–0.9999; 4.5–6.6), followed by the Sahlin–Peppas (0.9847–0.9880; 3.8–4.2), Higuchi (0.9633–0.9761; 2.8–3.4), Korsmeyer (0.9506–0.9819; 2.5–3.1, first-order (0.6709–0.8434; 0.87–1.5), and Hixon–Crowell (0.5645–0.6437; 0.60–0.95) models. 

Baker and Lonsdale proposed approximations for the complex equation that describes diffusional drug release from a spherical matrix system, when Sh ≫ 1, based on two different time scales. The early-time approximation (Equation (1)) holds for up to 40% of the release while the late-time approximation (Equation (2)) applies for 40–100% of the release [5].

(1) $$\frac{M_{t}}{M_{\infty }}=6{\cdot \left(\frac{D_{dif}\cdot t}{r^{2}\cdot \pi }\right)}^{\frac{1}{2}}-\frac{3\cdot D_{dif}\cdot t}{r^{2}}\;for\;0\leq \frac{M_{t}}{M_{\infty }}\leq 0.4$$

(2) $$\frac{M_{t}}{M_{\infty }}=1-\frac{6}{\pi^{2}}\cdot e^{\left(-\frac{{\pi^{2}\cdot D}_{dif}\cdot t}{r^{2}}\right)}\;for\;0.6\leq \frac{M_{t}}{M_{\infty }}\leq 1$$

where *M*t*/M*∞ is the drug fraction released at time *t*, *r* is the sphere radius, and *D_dif_* is the drug diffusion coefficient.

The theoretical curves aligned well with the experimental data plotted in Figure 4, with Equation (1) effectively describing the drug release up to approximately day 6. The drug diffusion coefficients obtained using the simplex algorithms and the least-squares minimization of the residuals are presented in Table 2, within a 95% confidence level.

**Figure 5:**
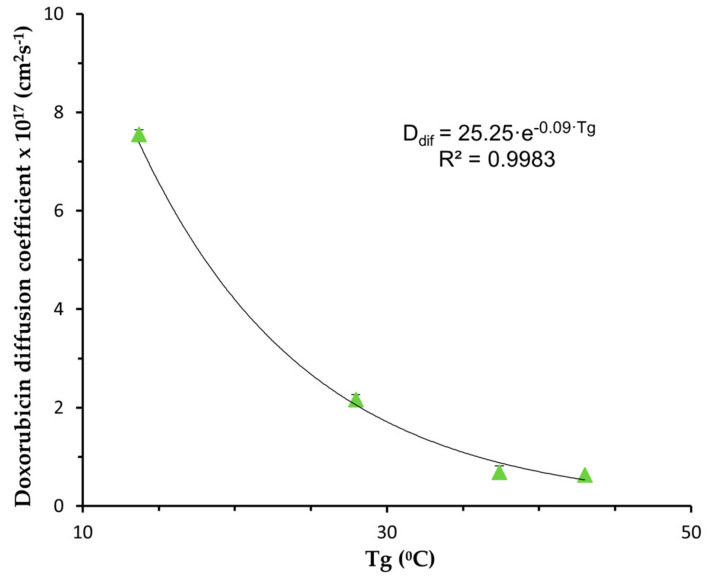
Doxorubicin diffusion coefficient as a function of polymer Tg. The solid line represents the exponential trend line fitting the data.

The obtained values of R^2^adj and MSC indicate that the release of doxorubicin from all tested NPs is controlled by a diffusion process. The drug diffusion coefficient values obtained ranged from 6.3 × 10^−18^ to 7.55 × 10^−17^ cm^2^s^−1^ and were in the same order of magnitude as those reported by Mu and Feng [27] for paclitaxel in PLGA NP and estimated by Arifin et al. [5] from published data, but much lower than those obtained by Jeong et al. [28] for ketoprofen in a PEG–PLGA–PEG triblock copolymer. It should be noted that the diffusion coefficient increases with an increase in PEG content in the polymer. This effect is subtle between PLGA and PLGA-PEG 5% NPs but becomes more pronounced as the PEG content increases to 10% and 15%. This behaviour can be attributed to the enhanced hydrophilicity of the polymer due to the presence of PEG, which promotes increased water uptake and subsequently augments the drug diffusion coefficient. Furthermore, as previously mentioned, an increase in PEG content leads to a decrease in polymer Tg, indicating greater flexibility in polymer chains that facilitates drug diffusion. Indeed, the impact of an increase in PEG content in the PLGA copolymer on Tg follows a similar trend, with a slight decrease observed, compared to PLGA for a PEG content of 5% and a more pronounced decrease for 10% and 15% PEG content. The dependence of the obtained diffusion coefficients on polymer Tg for doxorubicin-loaded NPs is illustrated in Figure 5, revealing an exponential relationship between these two parameters (r^2^ = 0.9983).

By substituting the equation depicting Tg versus % PEG (in Figure 3) into the exponential relationship between *D_dif_* and Tg, we can derive a relationship between the doxorubicin diffusion coefficient (*D_dif_*) and the percentage of PEG in the copolymer, which can be expressed as Equation (3).

(3) $$D_{dif}=25.25\cdot e^{\left(-0.09\cdot (42.9-0.09\cdot {\left(\%PEG\right)}^{2}-0.65\cdot \left(\%PEG\right)\right)}$$

Even though it is a practical equation, this equation holds significant utility as it enables the optimization of the doxorubicin release profile by adjusting the quantity of PEG in PLGA-PEG copolymers. This relationship serves as a valuable guide for experimental design in the development of delivery formulations, providing a structured roadmap for optimization.

### 3.3. Viability Assays

**Figure 6:**
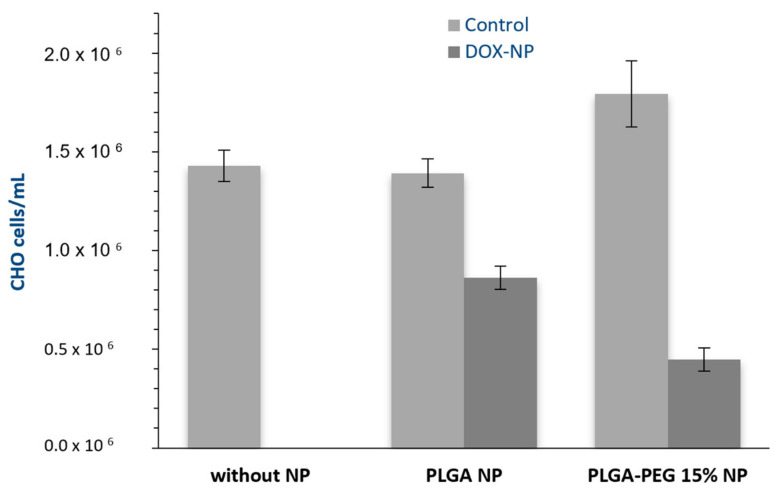
Absolute number of cells in experiments with the addition of 1 mg mL^−1^ of empty or DOX-loaded PLGA NPs and PLGA-PEG 15% NPs, (n = 3).

**Figure 7:**
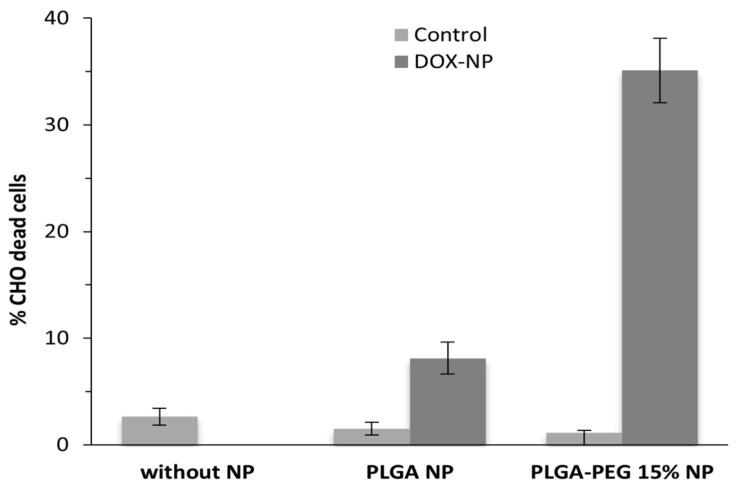
Percentage of CHO dead cells without NPs, with 1 mg mL^−1^ of unloaded or doxorubicin-loaded PLGA NPs and PLGA-PEG 15% NPs, obtained by flow cytometry (n = 3).

To investigate the effect of PEG presence in a PLGA polymer on in vitro toxicity, we cultured CHO cells in the presence of unloaded or DOX-loaded PLGA and PLGA-PEG 15% NPs for incubation periods of 72 h. Figure 6 and Figure 7 illustrate the effect of DOX-loaded NPs on both the absolute number of cells (determined by direct counting) and the percentages of dead cells (assessed via flow cytometry), respectively. The results for an assay without NPs added are also presented.

These results clearly indicate that the unloaded polymeric NPs tested are non-toxic and have a minimal impact on the number of viable cells. Furthermore, DOX-loaded PLGA-PEG 15% NPs exhibit a greater effect on cell viability loss, 75% vs. 38% for PLGA NPs, consistent with the observed drug release profiles (Figure 3), where they required less time than PLGA NPs to release a larger amount of DOX, thus demonstrating higher efficacy in inducing cellular death.

## 4. Conclusions

In this study, biodegradable DOX-loaded NPs of PLGA-co-PEG containing 5%, 10%, or 15% of PEG were successfully prepared using the o/w double-emulsion solvent evaporation method. The produced NPs presented a negative zeta potential and drug loadings ranging from 2.6 to 2.9 mg DOX/100 mg of NPs. The DOX release kinetics show a biphasic profile for all NPs, characterized by an initial burst release until day 16, followed by a gradual decrease in release rates thereafter. Among the mechanistic and semi-empirical models applied, the Baker–Lonsdale model provided the best fit for describing the drug release data, indicating that the drug release process is diffusion-controlled. The obtained DOX diffusion coefficients ranged from 6.3 × 10^−17^ cm^2^s^−1^ to 7.55 × 10^−17^ cm^2^s^−1^ and increased with increasing PEG content in the polymer, as expected, due to the greater hydrophilicity of the polymer provided by the presence of PEG as well as the decrease in polymer Tg which indicates enhanced flexibility in polymer chains that promotes drug diffusion. A mathematical relationship between the DOX diffusion coefficient and the percentage of PEG in the polymer was established, enabling the optimization of the doxorubicin release profile by modulating the PEG content in the PLGA-co-PEG polymer. In vitro tests conducted with CHO cells revealed that the unloaded NPs are non-toxic, while the DOX-loaded PLGA-PEG 15% significantly reduces cellular viability.

## Figures and Tables

**Table 1:** Zeta potentials, drug loading, encapsulation efficiency, and glass transition temperature of PLGA, PLGA-PEG 5%, PLGA-PEG 10%, and PLGA-PEG 15% NPs.

NP	Zeta Potential (mV)	DOX Loading (mg DOX/100 mg NP)	EE (%)	Tg (°C)
PLGA	−14.4 ± 0.8	2.9 ± 0.6	18 ± 4	43.1
PLGA-PEG 5%	−9.8 ± 0.7	2.8 ± 0.8	187 ± 5	37.4
PLGA-PEG 10%	−6.5 ± 0.7	2.6 ± 0.6	16 ± 4	27.9
PLGA-PEG 15%	−9.9 ± 0.9	2.8 ± 0.7	17 ± 4	13.7

**Table 2:** Drug diffusion coefficients obtained by the Baker–Lonsdale model.

NP	Diffusion Coefficient (cm^2^s^−1^)	R^2^_adj_	MSC
PLGA	(6.30 ± 0.13) × 10^−18^	0.9952	5.22
PLGA-PEG 5%	(6.88 ± 0.08) × 10^−18^	0.9999	6.62
PLGA-PEG 10%	(2.16 ± 0.03) × 10^−17^	0.9999	5.95
PLGA-PEG 15%	(7.55 ± 0.16) × 10^−17^	0.9989	4.52

## Data Availability

The data presented in this study are available on request from the corresponding author.
